# Algae as Nutritional and Functional Food Sources

**DOI:** 10.3390/foods12010122

**Published:** 2022-12-26

**Authors:** Fatma Boukid, Massimo Castellari

**Affiliations:** 1 ClonBio Group Ltd., D02 XE61 Dublin, Ireland; 2 Institute of Agriculture and Food Research and Technology (IRTA), Food Industry Area, Finca Camps i Armet s/n, 17121 Monells, Spain

Algae are a large and diverse group of autotrophic eukaryotic and photosynthetic aquatic organisms [1]. They can be divided into multi-cellular seaweeds, and unicellular microalgae (including cyanobacteria) [2]. In the last years, there has been a growing interest in algae as an essential part of the food of the future. Using the Scopus database (Elsevier’s abstract and citation database), a search was performed from 1990 to 2022, selecting “algae and foods” as keywords; a total of 17216 publications were obtained. Figure 1 shows that the literature growth rate is steadily increasing and follows an exponential model.

In the frame of the transition to plant-based diets, the use of algae as food ingredients has received considerable attention as a sustainable food source for human nutrition [1]. Compared to conventional plant- and animal-based sources, algae are highly productive and do not require arable/fertile land and do not need fresh water sources. Algae also may contribute into sixteen sustainable development goals of the 2030 agenda [3]. However, algal ingredients are mostly used as dry powder or derived products and not as a wet biomass. Considering that drying is a cost-intensive and energy-requiring process, innovative technologies such as indirect hybrid solar dryer was tested for drying microalgae (*Tetraselmis chui* and *Nannochloropsis oceanica*) wet paste [4]. Results revealed that the nutritional (total proteins, carbohydrates, lipids, and fatty acid profiles) and functional properties (solubility, water-, oil-holding, foaming and emulsifying properties) as well as the microbial safety of solar dried microalgae were comparable to those obtained from conventional methods (freeze or spray drying). Furthermore, the upscaling of solar dryer could contribute into reducing the cost of production and consequently boosting the market of algae ingredients beyond niche to mainstream.

As functional ingredients, algae offer a vast range of health-beneficial compounds such as protein, bioactive peptides, fatty acids, polysaccharides, sterols, minerals, and vitamins [5,6,7,8]. In a recent study, Afonso et al. [6] showed that the nutritional benefits of algae species can be further maximized through the modulation of the environmental conditions. Scientific evidence demonstrated several health promoting properties such as antioxidant, scavenging, reducing, and anti-proliferative activities in relatedness to algal phenolic and flavonoid contents [5]. Furthermore, based on in vivo trials, the combination of sulfated polysaccharide ulvan and the carotenoid astaxanthin promoted the relative abundance of *Bacteroidia*, *Bacilli*, *Clostridia*, and *Verrucomicrobia*, which are beneficial for intestinal health and gut homeostasis [9]. 

Thus, the use of algal ingredients is trending upward in commercial foods as reported by a recent market study [10]. It was found that the dominant algal ingredient is carrageenan owing to its thickening, emulsifying, stabilizing, and gelling features, while the main species used are *Chlorella* spp. and *Arthrospira* sp., ‘spirulina’ [10]. Recent research evidence revealed that microalgae can be successfully incorporated in vegetables creams [11], snacks [12] and pasta [13,14]. Boukid et al. [11] developed high-protein vegetable creams using single-cell ingredients from spirulina, *Chlorella vulgaris*, *Tetraselmis chui*, and *Nannochloropsis oceanica*. This addition (1.5 and 3%) improved the nutritional quality but impacted the physicochemical and rheological properties of the creams at different extent depending on the species and the level of addition. Creams made with 1.5% *C. vulgaris* were found comparable to the standard product (without microalgae). Bazarnova et al. [13] incorporated 5% of *Chlorella sorokiniana* in durum wheat pasta, which improved the nutritional quality (proteins, lipids, chlorophyll, and carotenoids) and provided a natural red color to the final product [13]. Compared to 100% durum wheat pasta, the incorporation of seaweeds (3% of *Ulva lactuca*, *Porphyra tenera*, and *Undaria pinnatifida*) improved the nutritional value and induced slight changes in the technological properties of enriched pasta [14]. To avoid nutrients deterioration during high temperature treatments, Tork et al. [12] used spirulina as a dragée for the coating of extruded corn snack. This dragée provided snacks with improved nutritional quality (flavonoids, anthocyanin, vitamins, protein, minerals, and fatty acids, including ω3 and ω6) and high sensory scores.

To conclude, even though the food market has strong motivations to include algae ingredients [2], the maximum inclusion level did not exceed 5% in most cases due to their peculiar color and umami taste (depending on the species) [11,12,13,14]. More research is deemed necessary for selecting new strains as well as developing technologies (e.g., purification and encapsulation) to enhance the organoleptic profile of algae and thus favor consumers acceptance. Furthermore, several bottlenecks including high production costs, health concerns related the toxicology and allergenicity of some species, and unclear legislation need to be addressed to boost unlocking the potential of algae [2].

## Figures and Tables

**Figure 1:**
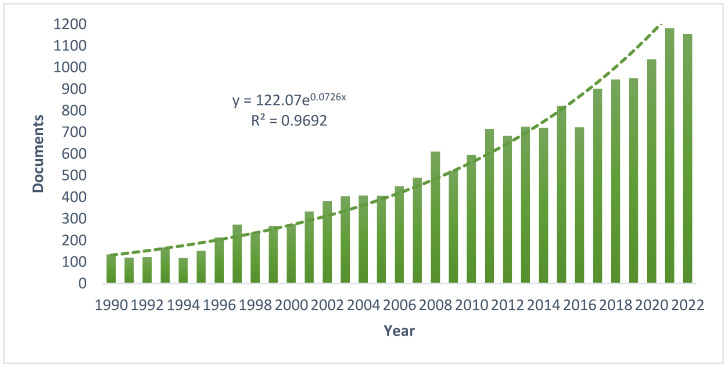
Evolution of the number of publications per year (from 1990 to 2022) reporting on algae as foods.
